# Determining the utility of diagnostic genomics: a conceptual framework

**DOI:** 10.1186/s40246-023-00524-1

**Published:** 2023-08-16

**Authors:** Andrew Mallett, Zornitza Stark, Zoe Fehlberg, Stephanie Best, Ilias Goranitis

**Affiliations:** 1 Australian Genomics, Murdoch Children’s Research Institute, Melbourne, VIC Australia; 2 College of Medicine and Dentistry, James Cook University, Douglas, QLD Australia; 3 Institute for Molecular Bioscience, The University of Queensland, St Lucia, QLD Australia; 4 Department of Renal Medicine, Townsville University Hospital, Douglas, QLD 4029 Australia; 5 Victorian Clinical Genetics Services, Murdoch Children’s Research Institute, Melbourne, VIC Australia; 6 University of Melbourne, Melbourne, VIC Australia; 7 Department of Health Services Research, Peter MacCallum Cancer Centre, Melbourne, VIC Australia; 8 Victorian Comprehensive Cancer Centre Alliance, Melbourne, VIC Australia

**Keywords:** Diagnostic genomics, Utility, Framework, Implementation, Ontology

## Abstract

**Background:**

Diagnostic efficacy is now well established for diagnostic genomic testing in rare disease. Assessment of overall utility is emerging as a key next step, however ambiguity in the conceptualisation and measurement of utility has impeded its assessment in a comprehensive manner. We propose a conceptual framework to approach determining the broader utility of diagnostic genomics encompassing patients, families, clinicians, health services and health systems to assist future evidence generation and funding decisions.

**Body:**

Building upon previous work, our framework posits that utility of diagnostic genomics consists of three dimensions: the domain or type and extent of utility (what), the relationship and perspective of utility (who), and the time horizon of utility (when). Across the description, assessment, and summation of these three proposed dimensions of utility, one could potentially triangulate a singular point of utility axes of type, relationship, and time. Collectively, the multiple different points of individual utility might be inferred to relate to a concept of aggregate utility.

**Conclusion:**

This ontological framework requires retrospective and prospective application to enable refinement and validation. Moving forward our framework, and others which have preceded it, promote a better characterisation and description of genomic utility to inform decision-making and optimise the benefits of genomic diagnostic testing.

## Background

Diagnostic efficacy is now well established for the application of diagnostic genomic testing in rare disease [1]. The impacts of this revolution in diagnostic care have been substantial both for individual patients and families, but also across health systems [2–4]. Achieving effective implementation within health systems can be complex and requires understanding health service utilisation patterns [5] in addition to contemporary assessments of broader utility. Given that the overwhelming number of studies reported to date of diagnostic genomics are short-term observational cohort studies rather than long-term randomised trials, the ability to assess utility in a comprehensive manner is impeded by the lack of control comparators as well as a focus on immediate outcomes such as diagnostic yield. We propose a conceptual framework to approach determining utility of diagnostic genomics to assist with further evidence generation and the development of more comprehensive models of utility with relevance across patients, families, clinicians, health services and health systems.

## Main text

### Previous work and frameworks

The need to define, systematically measure and value the utility of genomic testing has been recognised since the start of the transition of technology from research to clinical setting [6–9]. Valuations of genomic utility [10–13] and inclusion in economic assessments [14–16] have supported the understanding and delivery of patient-centred value-based implementation [17] and conceptualisation of the perceived benefits in clinical practice [18]. Attempts to standardise value measurement utilising existing literature with stakeholder refinement [19] has resulted in the development [20], and validation [21, 22], of a Clinician-reported Genetic testing Utility InDEx (C-GUIDE). The C-GUIDE seeks to capture the clinician perspective towards genetic testing utility relating to (1) understanding diagnosis and prognosis, (2) informing medical management, (3) awareness and actionability of reproductive and health risks for patients and family members, and (4) overall patient and family psychosocial well-being. Whilst clearly valuable, and a critical step towards robust and comparable measurement of genomic test utility, there is a need to embrace the multitude of contexts amongst both adult and paediatric testing scenarios [23], include patient reported outcomes [24, 25], and the diversity of clinical specialty fields in which genomic implementation is occurring [26]. Further, system-level decision-making, including but not limited to health technology assessments, necessitates other priority dimensions, such as cost-effectiveness, equity, budget impact [27], job creation and loss, and the value of genomic data for research and discovery. Importantly, we acknowledge that these do not all fall within a singular methodological approach to assessment [27]. Thereby, we build upon previous work to propose a broader conceptual framework to the potential measurement, assessment and valuation of genomic utility across multiple dimensions of relevance to patients, families, clinicians, health systems and economies.

### Genomic utility ontology

Our conceptual framework consists of three core utility dimensions, the domain or type and extent of utility (what), the relationship and perspective of utility (who), and time horizon of utility (when). Within each dimension a possible classification system is described which could be used to determine cumulative genomic utility.

#### Domain of impact (what)

The first dimension is the domain of utility which comprises of the type of utility and extent of impact (Table 1). The type comes in many potential forms that would include diagnostic, reproductive, therapeutic, prognostic, investigative, psychosocial and discovery/research (new knowledge generation) domains. Further exploring these domains, psychosocial impact is likely to be related to clinical dimensions but brings an additional “value of knowing”, highlighted as being valuable to individuals and families, even in the absence of associated changes in clinical management [10–12]. Previous studies [28] have described feelings of suffering having been legitimised, a sense of closure, and feelings of altruism, experienced against the backdrop of substantial prior uncertainty [29] even in the absence of associated changes in clinical management or a “remarkable” finding [30]. Within the types of utility, there might be a variety of relative measures across a continuum from a minimal identifiable level of impact to maximal impact. Given the need to apply a standardised framework that enables reproducible measurement, qualitative levels or statements may assist in the definition of measures. For example, the extent of impact on the ‘diagnostic’ domain could be defined from (+) clarified an existing clinical diagnosis, to (++) changed an existing clinical diagnosis, or (+++) provided a new genomic diagnosis that could not have been reached by other investigations. We expect through application and revision of the framework that future iterations will include refinement of domains and measures.

**Table 1:** Domain of impact (what)

Extent of impact *with example measures*			
Type of utility	Low impact	Moderate impact	High impact
	(+)	(++)	(+++)
(i) Diagnostic	*Clarified* diagnosis	*Changed* diagnosis	*New* diagnosis
(ii) Reproductive	Information *provided*	Information *used*	Reproductive outcome *altered Completed*
(iii) Therapeutic	*Avoided* therapy	*Altered* existing therapy	*New* therapy
(iv) Prognostic	*General* information	*Clarified* information	*Precise* information
(v) Investigative	Avoided *simple* investigation	Avoided *complex* investigation	Avoided *invasive* investigation
(vi) Psychosocial	Certainty/self	Belonging/support	Empowerment
(vii) Discovery	Clarified	Changed	*New* treatment developed
*To be defined/added*			

Types and extent of impact with example measures

#### Relationship and perspective of utility (who)

The second dimension in which all the domains of utility can be considered, is the frame of personal, family and community (Table 2) context. For instance, a specific utility might relate to one’s self, an immediate family member, a more distant family member, the community of that individual and/or family, or indeed society as a whole. Where a diagnostic genomic result might have utility for one’s self diagnostically, this same result may be of demonstrable benefit to an immediate family member through cascade testing, or for a more distant family member in terms of whether they might be a living related kidney donor, and to the community more broadly as it might result in new knowledge that results in a novel treatment or clinical trial. Within a broader conceptualisation of “community” would also be additional third-party stakeholders such as clinicians, health payers and researchers whose perspectives would be critical for policy purposes.

**Table 2:** Relationship and perspective of utility (who) and time horizon of utility (when)

Timeframe to impact				
Relationship of Impact	a. Immediate (< 6 months)	b. Short (6–24 months)	c. Medium (2–5 years)	d. Long (> 5 years)
1. Self	1a	1b	1c	1d
2. Family unit and 1st degree relatives	2a	2b	2c	2d
3. ≥ 2nd degree relatives	3a	3b	3c	3d
4. Community	4a	4b	4c	4d

“a-d” corresponds to the 4 vertical columns representing the 4 time periods

#### Time horizon of utility (when)

The experience, observation or perception of utility can also be described in terms of its timing (Table 2). The timing of benefit impacts the level of valuation, forming an interplay with people's inherent discounting of future gains. For instance, in Australia future costs and benefits are discounted by an annual rate of 5% in reflecting that a benefit in the future has less value than the same benefit today [31]. Potential examples of time horizons of utility might represent immediate avoidance of alternate invasive non-genomic investigation, short term access to directed or targeted treatment, and medium-long term utility from reproductive planning.

Additionally, evidentiary uncertainty across timespans may impact priorities and values. Where some forms of utility might be experienced in the immediate or short-term, others might be more distant or very long term. Whilst this might take the form of describing when such utility occurs or how long it occurs for, in the first instance we propose to seek inclusion of the former with potential to include the latter as our conceptual framework evolves and is tested. Though there may be some differences in relative surety or precision of utility across diverse incident timepoints and for different items being examined, this should not preclude inclusion given so long as the item’s existence is considered to be actual or potential beyond reasonable doubt. As such, we propose a third dimension of utility measurement across differing time horizons to ensure that this is captured.

### Cumulative and preference-based valuation of utility

**Fig. 1:**
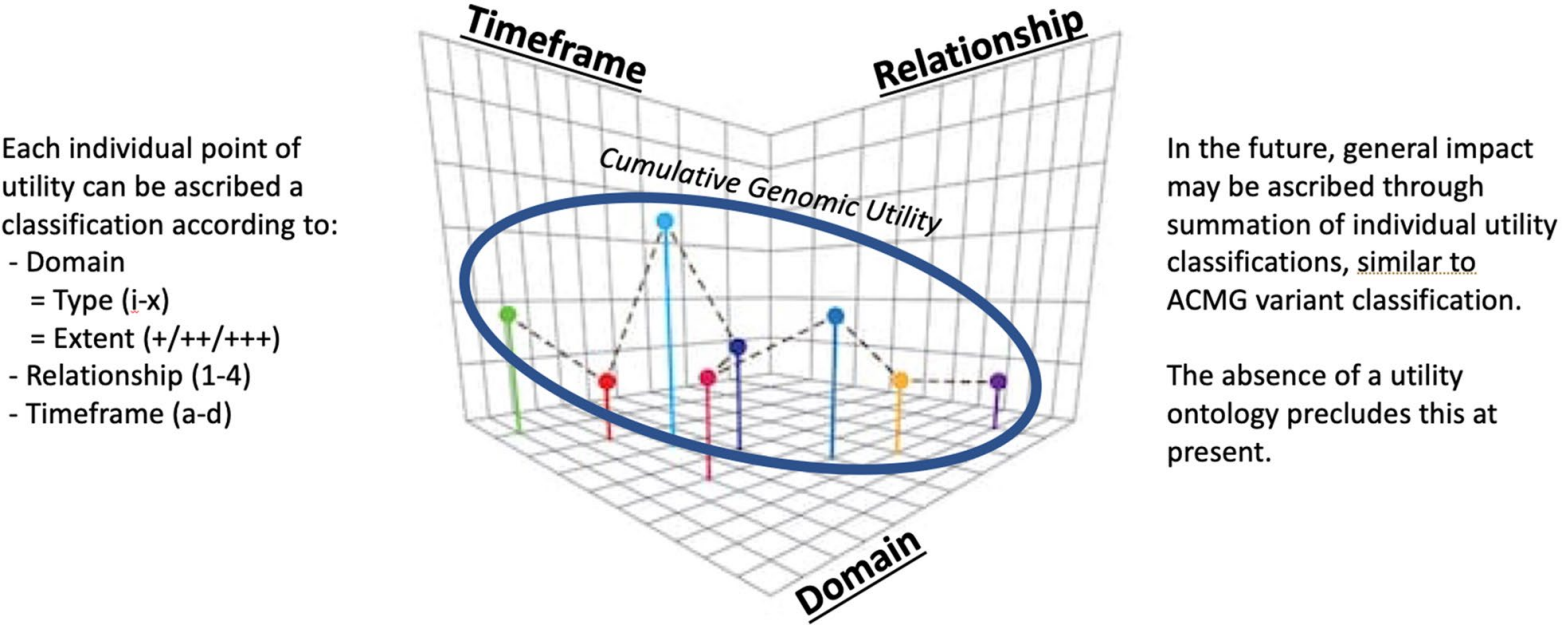
Proposed framework for description, assessment and summation of genomic utility

Across these three proposed dimensions of utility of what, who and when, one could potentially triangulate a singular point of utility axes of type, relationship, and time (Fig. 1). Collectively, multiple different points of individual utility might be inferred to relate to a concept of aggregate utility. Whether and the extent to which such a cloud effect is quantitatively or qualitatively approached should be the source of subsequent observation, experimentation, and analysis. Nevertheless, an understanding of the shape, size, and characterisation of a collective utility would be useful for understanding current and future interventions. For policymakers and those influencing complex systems, such as health systems, this would enable a more level playing field upon which to compare different potential interventions that might generate utility, and facilitate equitable implementation followed by informed audit & quality assurance evaluation. At present, there is a very real risk of biasing comparisons or value interpretations within a system that has limited resource boundaries, with resultant threats to generalisable community benefit as new interventions are evaluated for implementation upon an unknowingly asymmetric landscape. An attempt to describe aggregate genomic utility for such interventions that are being considered for implementation may make this endeavour more transparent and equitable whilst having positive effects on public policy and economy.

### Future approaches to refine, value, and implement

Initial steps to begin understanding such a multidimensional and aggregate description of genomic utility might incorporate retrospective alignment to existing interventions under evaluation, such as diagnostic genomic testing in different rare diseases. Apart from attempting to validate such an approach, this would also seek to undertake two major activities that require real-world framing. Firstly, does the population contain as full as possible representation of utility types and diversity to accompany the range of potential individuals that utility aligns to and the time horizons it might be experienced in. Secondly, a discussion should ensue as to potential quantification of utility. One such framework might see different aspects of a point utility accruing different criteria which can be added together combinatorically to align to tiers of utility, while reflecting the priorities of the key stakeholders involved (Fig. 1). This framework would be somewhat similar to the current approach to genetic variant classification using the accumulation of various different American College of Medical Genetics and Genomics variant criteria or extend to multi-attribute utility theory [32]. In that way, different of interventions can be meaningfully compared to inform health care prioritisation.

Whichever approach might emerge from initial retrospective application and refinement, we propose that the genomic utility ontology might then begin to be incorporated into prospective studies of interventions, including cohort studies and randomised clinical trials. Pilot work might look at policy implementation within existing frameworks for evaluation and implementation of public funding or licensing of health interventions such as the broader implementation of clinical genomics in health systems, especially those underpinned by universal health care.

### Major challenges and limitations

There are naturally many challenges to approaching a broadly applicable ontology to measuring utility of genomic testing. One is that any approach informed by existing literature is naturally limited by that literature in terms of not appreciating aspects of utility that are not measured, described or presented. Utility might also have different perspectives according to different values, sociocultural norms and health system structures. Our proposed multidimensional conceptualisation of utility description might also prove either too granular or too opaque for different groups of patients, clinicians or policymakers. Further, identifying an appropriately broad and descriptively reported series of past research cohorts upon which to build and validate this ontology is likely to generate substantial activity requirements. Nevertheless, and in spite of these challenges, the likely opportunity cost of not undertaking efforts to move iteratively towards a comprehensive genomic utility framework would be far greater than would need to be absorbed by undertaking such activities, ensuring the equitable and sustainable translation of genomics.

## Conclusions

In summary, we propose a conceptual framework for the potential measurement, assessment, and valuation of genomic utility that builds from previous work whilst evolving a multidimensional construct that contextualises utility both now and into the future. This ontological framework requires application both in retrospective cohorts as well as in prospective studies to enable its refinement and potential validation. The relative values and preferences of different stakeholders across a continuum of genomic utility scenarios also requires further study. Moving forward this framework, and others which have preceded it, will continue to promote a better characterisation and description of genomic utility such that personal, local and system decision-making can be best informed to derive optimal benefit from genomic diagnostic testing.

## Data Availability

Not applicable.

## Abbreviation

C-Guide: Clinician-reported Genetic testing Utility InDEx
